# Supplementary material for: Neural network based 3D tracking with a graphene transparent focal stack imaging system
Source: Nat Commun. 2021 Apr 23;12:2413. doi: 10.1038/s41467-021-22696-x (PMC8065157; doi:10.1038/s41467-021-22696-x)
Supplement: Supplementary file 1 — Supplementary Information [file 41467_2021_22696_MOESM1_ESM.pdf]

Supplementary Information

Supplementary Information 1: System Hardware

I. Graphene Transparent Photodetector Device Fabrication:

We first transferred a layer of graphene onto a commercial glass substrate. The graphene layer was grown on copper foil with chemical vapor deposition (CVD), and a standard wet transfer process produced a decent coverage of the monolayer graphene on the centimeter scale[1]. We patterned the graphene layer into isolated squares (as the floating gate) using photolithography. Then we etched away the exposed graphene with oxygen plasma. A 6-nm layer of sputtered silicon was sputtered on top of the graphene layer as the tunneling barrier. Another layer of graphene was transferred on top of the barrier immediately after silicon sputtering to minimize surface oxidation. We annealed the sample in Ar at 300 C for 15 mins to enhance graphene's adhesion with the substrate, so that there is less stripping-off in the subsequent process. This graphene layer was lithographically patterned into the channel of the phototransistor as well as interconnects. The individual device pixels were spaced 0.3 mm away from each other. Cr/Au metal contacts were then deposited and connected to the graphene interconnects, leaving a 2.5 mm by 2.5 mm transparent window for light to pass through. The Cr/Au pads were then wire bonded to a sample holder in the readout circuit. The readout circuit was a scanning line that selectively applies bias to different pixels and collects the photocurrent in the target pixel.

II. Graphene Photodetector Characterization:

The fabricated all-graphene devices in general showed good coverage over the entire die. To check the uniformity and device yield, we applied a 0.5 V bias voltage across every pixel and measured the current across the graphene channel (see Supplementary Fig. 1(c)). Of all 192 devices tested, only 2 devices showed open circuit. This DC conductance test result shows 99% graphene device yield and negligible graphene peeling-off during the nanofabrication process.

The detector photoresponse is characterized using a 532 nm Verdi V10 CW laser under 0.22 μW illumination on a single pixel. For the responsivity calibration of both detector layers, we first align the lateral position of the imaging chip to maximize the photocurrent readout from the center pixel. This ensures that the optical beam is centered on the chip. Then we move the lens of the camera system along

the optical axis to provide a beam spot with a diameter > 4 mm. The large spot size provides nearly uniform illumination in the 0.9-mm-wide detector array. The beam sizes are measured using a power meter and a blade as the moving mask. Then we measure the photocurrent from the array. By calculating the illumination power per device area, we calculate the responsivities of the devices.

The responsivity is 3 A/W under 0.5 V source-drain bias. It is slightly smaller than previously reported values due to a relatively small bias voltage applied, lower doping level of graphene, and the geometry of the device. The DC photocurrent shows both a fast response on the scale of seconds and a slow response in hundreds of seconds, which is due to the charge trapping effect of the highly defective silicon barrier. By changing the dielectric material, the response time of such a structure can be decreased to the sub-millisecond level[2].To increase the speed of measurement and remove effects from drifting dark currents, we adopted an AC photocurrent measurement scheme to measure the smaller but fast component, as discussed in the main text. Supplementary Fig. 1(e) shows a linear dependence of AC photocurrent with respect to illumination power. A linear power dependence of the AC photocurrent was observed.

We also characterize the transparency of the graphene detector array with a light focused on the detector plane. Transmission of a 532 nm laser beam through the array is measured to be 81%, while the reflection of the uncoated glass substrate contributes to a transmission of 86%, as measured in the graphene-free area of the device. The graphene detector array contributes to only 5% of decrease in transmission. If the application requires, the transparency can be significantly improved to >95% using an antireflection coating, and by replacing the silicon layer with ALD-grown Al_2O_3 of the same thickness.

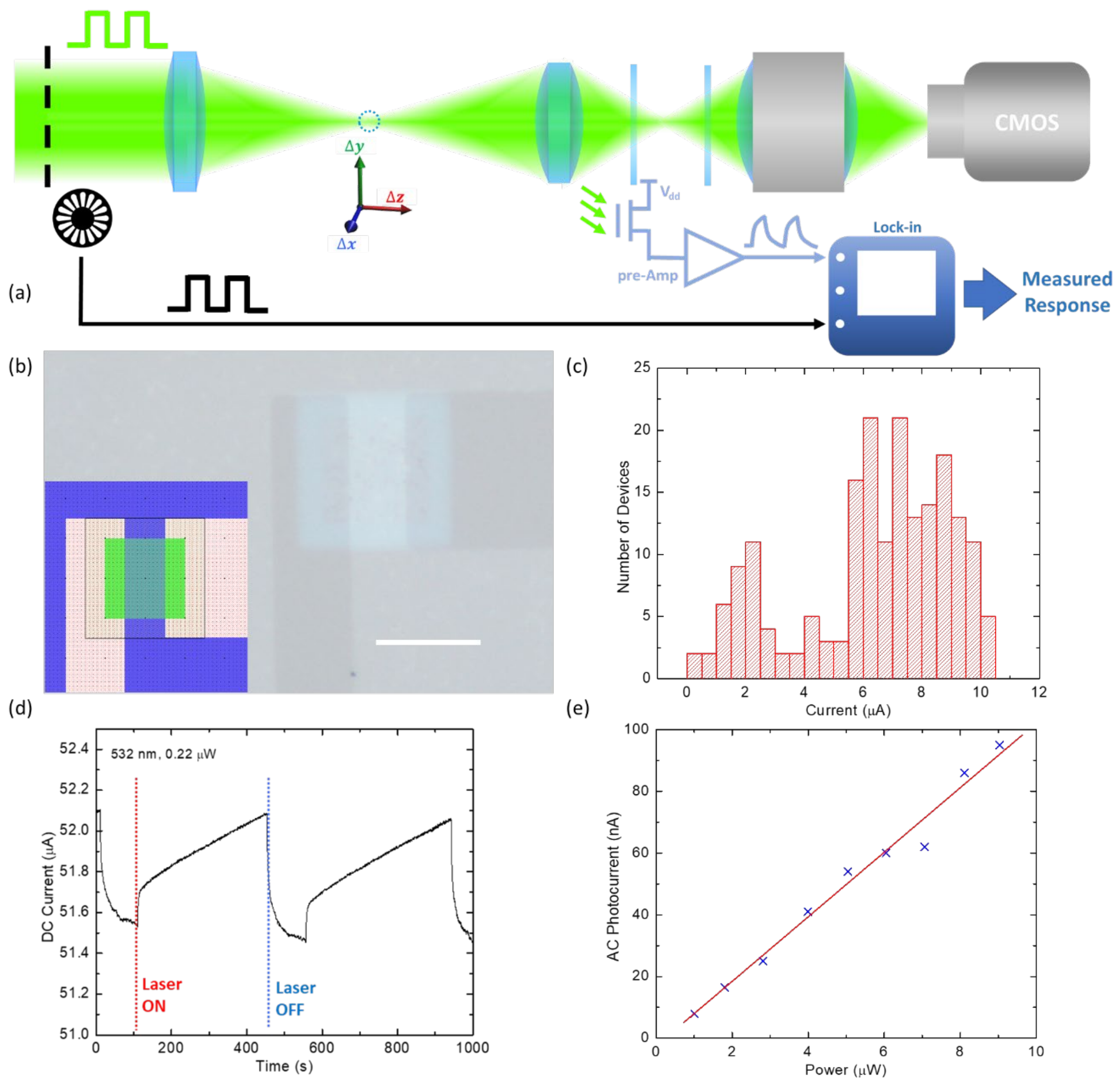

Supplementary Figure 1. Optical and electrical measurements on the all-graphene transparent photodetector. (a) measurement setup. A point object (dotted circle) is generated by focusing a green laser beam (532 nm) with the lens. Its position is controlled by a 3D motorized stage. Two detector arrays (blue sheets) are placed behind the lens. An objective and CCD camera are placed behind the detector array for sample alignment. A chopper modulates the light at 500 Hz and a lock-in amplifier records the AC current at the chopper frequency. (b) Optical microscope image and layout diagram (inset) of a single pixel. Blue: top layer graphene channel; green: bottom layer floating gate. The overlapped channel region (separated by the tunneling barrier) is 30 μm by 10 μm. The lower floating gate layer is 20 μm by 20 μm, intentionally made larger to avoid peeling-off. Scale bar: 20 μm. (c) Histogram of the DC currents across graphene channels for individual detector devices. Bias voltage applied is 0.5 V. (d) DC temporal photoresponse of a typical graphene detector following light illumination. Both a fast and a slow

component were observed, while the background current also showed drifting over time. (e) Power dependence of AC photocurrent, which measures the fast component and suppress the background drift. A linear power dependence (red line) is observed.

III. Use of Graphene for Interconnects

In our transparent detector array design, graphene is used not only as the active pixel material but also as the passive interconnect. Compared with other transparent electrode materials, such as indium-tin oxide (ITO), graphene is atomically thin while maintaining similar conductivity. This ultimate thin-ness minimizes optical interference patterns generated from the interconnect patterning, and also suppresses edge scatterings from normal metal wires. Supplementary Fig. 2 compares the optical transmission images of detector arrays fabricated using graphene interconnects versus ITO interconnects under 532 nm laser illumination. Even though the interference and scattering effect from ITO interconnects can be reduced with a refractive index compensation layer, this would add more complexity to the sensor array.

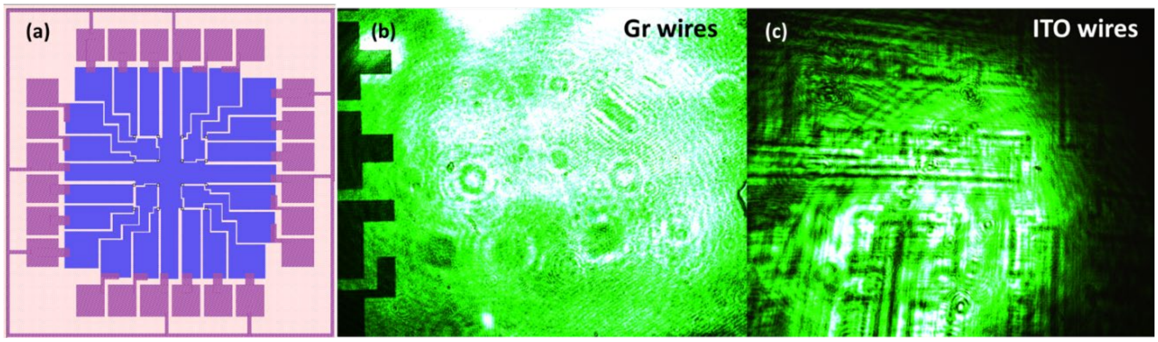

Supplementary Figure 2. Array design (a) and optical images of photodetector arrays captured by CMOS camera. Samples using graphene interconnects (b) showed significantly weaker effects of interference and scattering than samples with ITO interconnects (c).

IV. Readout of Single Pixel in 3D Ranging

Before using the graphene devices as an imaging array, an imaging hardware reliability test is performed (Supplementary Fig. 3). The single-pixel photocurrent is measured while the light source moves both in-plane and along the optical axis. For each pixel tested, the photocurrent always shows a single peak when the object is translated in 3 dimensions. The peak positions and FWHMs of peaks match the relative positions of pixels tested, confirming that the graphene detector array can indeed accurately "image" the focus of a point light source.

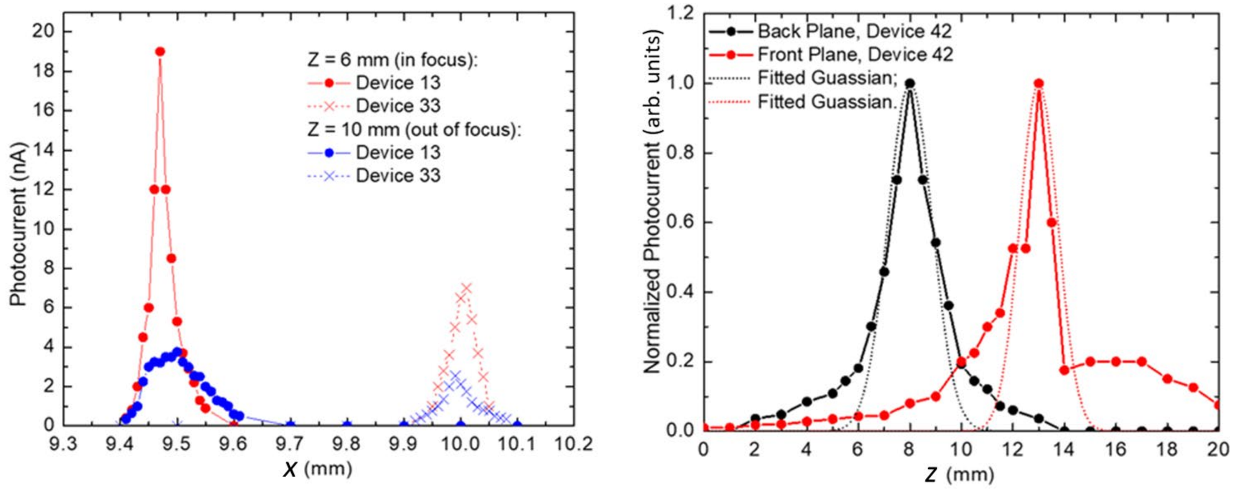

Supplementary Figure 3. 3D ranging test of single detector pixels. Left: Photocurrents from two pixels (Device 13 and 33) spaced laterally in the *X* direction. When the point light source moves in *X* direction, the devices are illuminated sequentially and give peaks at two different *X* positions. When the point source is off focus on the device plane, a broader peak is observed corresponding to de-focusing. Right: Photocurrents from two pixels at front and back planes along the optical path (*Z* direction), respectively. As the point source moves from front to back along *Z* direction, the focus shifts from front to back detector plane accordingly, resulting in the observed photocurrent peaks at different *Z* positions. Deviation from the Gaussian fit stems from imperfect beam quality and possible off-axis alignment.

V. Noise Analysis

The noise equivalent power (NEP) is a good measure to discuss the SNR in realistic applications. The NEP of the device has been discussed in the supporting information of our previous work (Liu C H, et al. Nature Nanotechnology, 2014, 9(4): 273-278.). We collect our data with a modulation frequency of 500 Hz. At this frequency, the noise spectral density is 10^{-9} A/Hz$^{1/2}$. The noise level is consistent with the $1/f$ noise of graphene transistors observed[3]. This indicates that the channel's $1/f$ noise dominates over the shot noise of dark currents in the tunneling barrier. With an AC responsivity of 10 mA/W (Supplementary Fig. 1 (e)), the NEP is 0.1 μW/Hz$^{1/2}$. The value is small compared with our test illumination power of ~10 μW per device.

We can also compare this with realistic illumination powers in a camera system. Assume a camera system with a 20-mm aperture and a numerical aperture of 0.7. When using it to image a white Lambertian

surface under sunlight, the estimated optical power per pixel is 0.05 μW. This indicates a relatively low SNR for our current device.

The low SNR is largely due to the slow response of our photodetectors, which is caused by the large density of charge traps in the tunneling barrier. Charge traps capture the tunneling charges and compensate the local field that motivates more interlayer hopping. One of our previous work replaced amorphous silicon with high quality Al_2O_3. The responsivity at 1 kHz is as high as 60 A/W at 532 nm[2]. Taking all the corresponding design variations, including increased noise due to a larger channel current, we expect a NEP of 0.1 nW/Hz$^{1/2}$, which is more than enough for realistic applications. In this experiment, we did not adopt the Al_2O_3 barrier due to fabrication yield considerations, as the thin material is vulnerable to the base used in lithography. Nevertheless, there are no fundamental limitations that prevent us from fabricating transparent devices with higher speed and responsivity.

In the above discussion, experimental results suggest that the tunneling current is not the major contribution of noise. For a more complete discussion, we can further analyze the tunneling noise's order of magnitude. The shot noise's current spectral density is $S = 2eI$ when the interlayer bias $V >> kT/e$ [4]. The current is the total of the dark current and the photocurrent, which is around 10 pA in our device. Hence the noise current density of the tunneling photodiode (before amplification) is around 1.8 fA/Hz$^{1/2}$. The value is much smaller than the photocurrent at any realistic illumination power. Also notice that $1/f$ noise is not considered here, so that the estimation only sets the lower limit for the noise amplitude contributed by the tunneling current before amplified with the photogating effect.

Moreover, neural networks can be trained to be robust against input noises. This further lifts the SNR requirements for the reported application.

In conclusion, the device's noise is dominated by the $1/f$ noise in the channel. The photoconductive gain amplifies the noise from the vertical tunneling diode. However, it does not dominate the device noise based on both tests and order-of-magnitude estimation. Better implementation of the device to image ambient objects needs an increase in responsivity. One promising way is to improve the tunneling barrier quality, which is also supported by previous work.

Supplementary Information 2: Data Processing and Machine Learning

I. Single-Point Object Focal Stack from CMOS Camera

We recorded 1,331 single-point object focal stacks using the transparent graphene transistor array and separately using a CMOS sensor (Thorlab DCC1645C); see the right part of the Fig. 2(a) in the main text.

By moving the CMOS sensor along z to focus either closer to or farther away from the lens, we captured focal stacks from CMOS camera. This data allows us to test how the image resolution and image quality of the graphene sensors affect the 3D ranging performance of a machine-learning algorithm.

We applied the following procedure to each high-resolution (1280 × 1024) color image captured by the CMOS camera: we convert the captured color image to gray image and optionally smooth it by spatial averaging and generate low resolution single-point object focal stacks of spatial size 4 × 4, 9 × 9 or 32 × 32. We used the processed images in either single-point tracking (to investigate the effects of imaging resolution to the tracking performance) or synthesizing multi-point object focal stacks.

II. Synthesizing Multi-Point Object Focal Stack

We synthesized multi-point object focal stacks by combining focal stacks from the scanned single point object (either from transparent graphene transistor array or from CMOS camera). The synthesis assumes that the detector's response is linear, i.e., suppose I_i is the sensor image of the single point object at location (x_i, y_i, z_i). Then the sensor image I_{multi} consisting of multiple points is synthesized as $I_{\text{multi}} = \sum_{i=1}^{N} I_i$, where N is the number of point objects.

We constructed an M-point object focal stack dataset, where the dataset consists of multiple subsets, and each subset consists of K possible shapes (relative position between points), by synthesizing each shape independently and then combining them. We translated an object to all possible locations (i.e., no point of M-point object is off the 3D grid) in the 3D $11 \times 11 \times 11$ scanning grid; at each location, we synthesize the corresponding focal stack according to the summation above. The number of synthesized datasets with ($M = 2$, $K = 2$), ($M = 2$, $K = 3$), ($M = 3$, $K = 2$), ($M = 3$, $K = 3$) were 1600, 2320, 1232, and 1880, respectively.

We constructed the rotating 2-point object focal stack dataset by selecting focal stacks from the M-point focal stack with K possible shapes dataset, with $M = 2, K = 4$. Four shapes of a 2-point object (i.e., $M = 2, K = 4$) were chosen to have same inter-point distance but rotated by different angles (26.5°, 63.5°, 116.5°, and 153.5°) about z axis (e.g., (1,0,0) means 0° rotation about $z$axis and (0,1,0) means 90° rotation about $z$axis). To form the helical trajectory in the $M = 2, K = 4$ setup, we selected an angle from the set {26.5°, 63.5°, 116.5°, and 153.5°} at each z position in the following sequence: 63.5°, 26.5°,153.5°, 116.5°, 63.5°, 26.5°, 153.5°,116.5°, 63.5°, 26.5°, 153.5°, for z =-10 mm, -8 mm, ..., 10 mm. See graphical illustration in Fig. 3(e) of the main paper.

III. Extended Object Focal Stack

We captured extended object focal stacks using the CMOS sensor. The experimental setup is shown in Supplementary Fig. 4. We used a ladybug as the extended object and moved it in a 3D spatial grid of size 8.5 mm × 8.5 mm × 45 mm. The grid spacing is 0.85 mm along both x and y, 3 mm along z. At each grid point, the object has 8 possible orientations in the x-z plane, with 45° angular separation between neighboring orientations. This led to a total of 1,5488 focal stacks, where each focal stack consists of two images captured by the CMOS sensor positioned at different z positions. Similar to Part B-III, all images are converted to gray images before feeding to the neural networks.

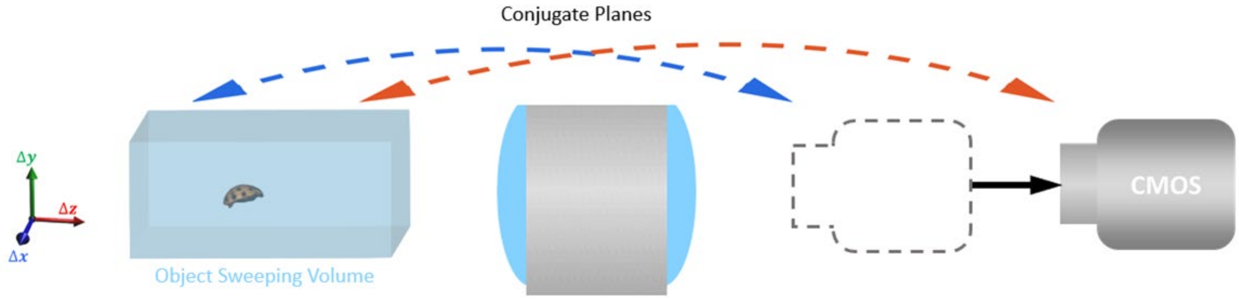

Supplementary Figure 4. Experimental set-up for capturing the extended object (ladybug) focal stack, using CMOS sensor.

IV. Neural Network Architectures and Training

We implemented all neural networks in Pytorch (ver. 1.0). The network architectures and training details are described below.

For single-point object tracking, separate neural networks were trained for estimating the three spatial coordinates x, y and z, respectively. Supplementary Fig. 5(a) shows the network architecture used for estimating coordinates x and y, and Supplementary Fig. 5(b) shows the network architecture used for estimating z. For multi-point object tracking, a single neural network (Supplementary Fig. 5(c)) is trained to estimate all points' coordinates.

In point object tracking cases (Supplementary Fig.5 (a-c)), the focal stack data is flattened into a one-dimensional vector and subsequently passed through multilayer perceptron (MLP)[5] using Rectified Linear Unit (ReLU) as the activation function.

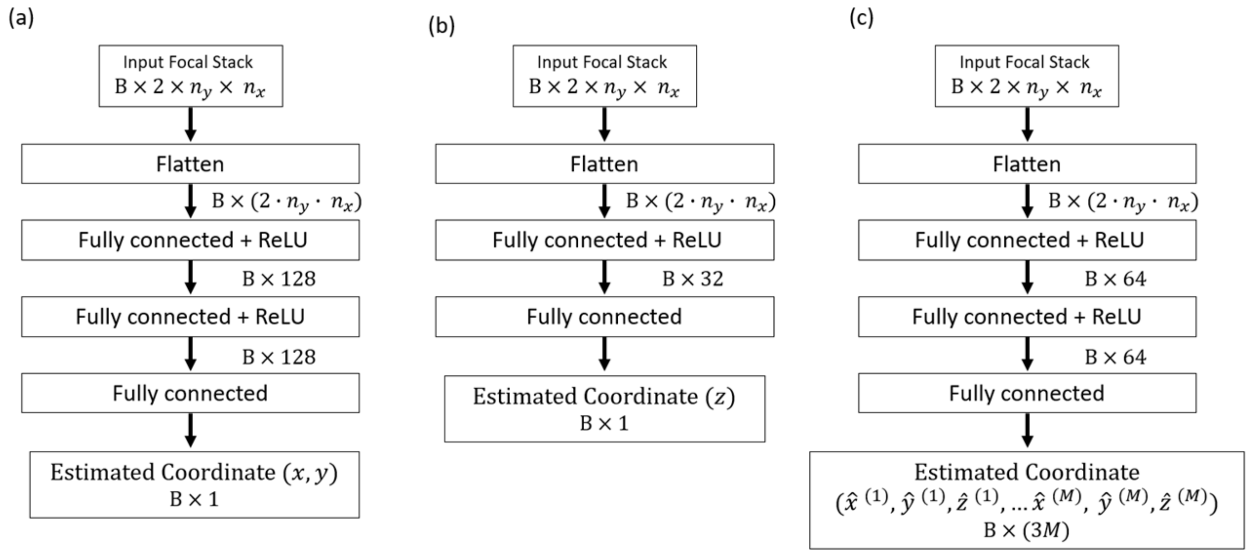

Supplementary Figure 5. Neural network architectures for 3D ranging. B is the general batch size of the data (e.g., in training, B is the training batch size; in testing with a single sample, $B = 1$). (a) Network for estimating single point object's x or y coordinate. (b) Network for estimating single point object's z coordinate. (c) Network for estimating M-point object's (x_i, y_i, z_i) coordinates tuple.

For single-point object tracking, the network outputs a single coordinate value for each focal stack, and the networks are trained by minimizing the following mean-square error (MSE) loss

$$\frac{1}{N}\sum_{i=1}^{N}\left(\hat{s}_i - s_i\right)^2,$$

where N is the number of training samples, s_i is the true spatial coordinate (x_i, y_i, or z_i) and $\hat{s}_i$ is the estimated spatial coordinate from a neural network. We trained networks using the Adam[6] optimizer with the learning rate of 10^{-2}, the training batch size of 50, and 2000 epochs.

For training multi-point object tracking neural networks, we defined the following MSE loss that considers the ordering ambiguity of the network outputs in training:

$$\frac{1}{N}\sum_{i=1}^{N} \min_{(p_1,\dots,p_M)\in P} \sum_{j=1}^{M}\left(\hat{x}_i^{(j)} - x_i^{(p_j)}\right)^2 + \left(\hat{y}_i^{(j)} - y_i^{(p_j)}\right)^2 + \left(\hat{z}_i^{(j)} - z_i^{(p_j)}\right)^2, \tag{1}$$

where M is the number of points of the object, P is the set containing all possible permutations of the tuple $(1,2,\ldots M)$, $x_i^{(j)}$ and $\hat{x}_i^{(j)}$ are the true and estimated coordinate of the ith data sample, j^{th} point. The network outputs a coordinates tuple for all the points of the object as $\{(\hat{x}^{(1)},\hat{y}^{(1)},\hat{z}^{(1)}),\ldots,(\hat{x}^{(M)},\hat{y}^{(M)},\hat{z}^{(M)})\}$. To consider the ordering ambiguity of the network outputs in training, e.g., for $(x^{(1)},y^{(1)},z^{(1)})$, the network cannot determine which estimate gives lower MSE, between $(\hat{x}^{(1)},\hat{y}^{(1)},\hat{z}^{(1)})$ and $(\hat{x}^{(2)},\hat{y}^{(2)},\hat{z}^{(2)})$, we found proper orders by minimizing MSE over the permutation set P in (1) . With the help of minimization over P, the loss will be low as long as a trained network predicts the overall shape of the object, regardless of the order of the network estimates. In the training, we scaled down the true z coordinate values by 33.3 so that it is in the same range as coordinates x and y. This avoids the loss (1) from being dominated by z component of MSE loss, i.e., avoids training from being biased to z-coordinate estimation. We trained the network using Adam optimizer with the learning rate of 10^{-3}, the training batch size of 100, and 2000 epochs.

For tracking the two-point rotating object, we also trained the network by (1) and scaled the z coordinate values by 33.3. For training the network, we used Adam optimizer with the learning rate of 10^{-3}, the training batch size of 100, and 2000 epochs.

For extended object tracking and orientation estimation, we use two convolutional neural networks (CNNs) (Supplementary Fig. 6) similar to VGG-16[7]. The CNN shown in Supplementary Fig. 6(a) is used for the tracking. For each focal image, we first extract high-level feature maps with multiple convolution-batch normalization (BN)-ReLU-pooling layers. Then we apply the following procedure to extracted feature maps from all focal images: 1) concatenation of all feature maps along channel dimension, 2) average pooling, 3) flattening, and 4) feeding the output into fully connected layers (FC) that lead to final coordinates. The network is trained by minimizing the following MSE loss:

$$\frac{1}{3N}\sum_{i=1}^{N}\left(\hat{x}_i - x_i\right)^2 + \left(\hat{y}_i - y_i\right)^2 + \left(\hat{z}_i - z_i\right)^2,$$

where N is the number of training samples. In the training, we scaled the true z coordinate values to have the same range as x and y coordinates, for the same reason as in the multi-point object tracking. The CNN shown in Supplementary Fig. 6(b) is used for object orientation estimation. We consider the problem as a multi-class classification problem: the CNN takes focal stack as input and output scores that are used to classify the object orientations with eight different orientations. The network is trained by minimizing the cross-entropy loss.

We trained both CNNs with 13,188 training samples, using Adam optimizer with the initial learning rate of 10^{-4} (with learning rate decay by 0.3 at epoch 3, 5, 10, 20), the training batch size of 20, and 60 epochs and tested trained CNNs with 2,300 test samples. Due to the nondeterministic behavior of PyTorch[8], the training/testing is repeated three times for the orientation classification network and the best model is used. The orientation classification accuracies of the three runs are 95.35%, 96.61% and 99.35%.

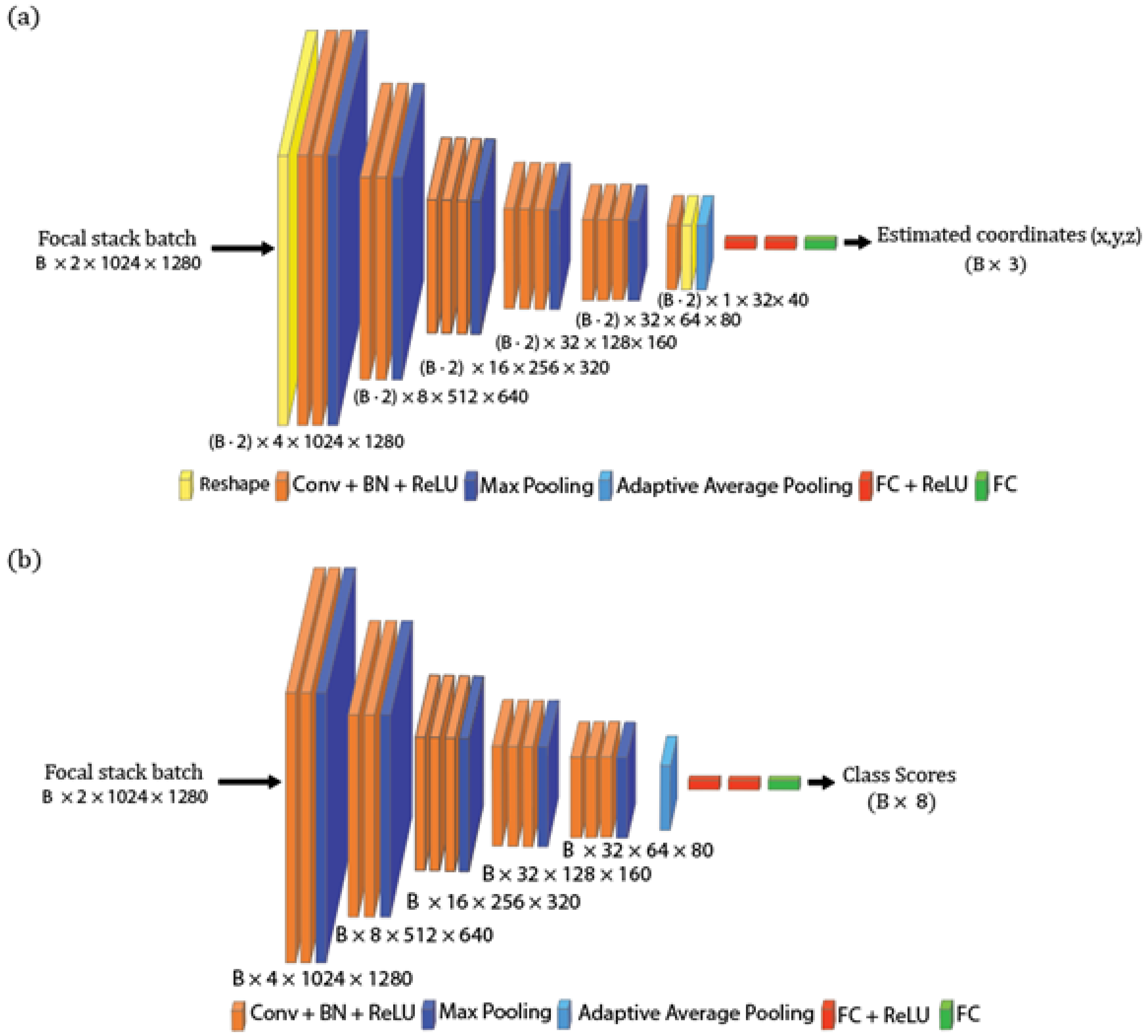

Supplementary Figure 6. Convolutional neural network architectures for extended object tracking and orientation estimation. B is the general batch size of the data (e.g., in training, B is the training batch size; in testing with a single sample, $B = 1$). (a) Network for estimating extended object's spatial coordinates (x, y, z). (b) Network for estimating extended object's orientation.

V. Ranging Performance Comparison

We studied the effect of the detector resolution and spatial smoothing on the single-point object 3D ranging performance. Supplementary Table 1 summarizes the results. The resolution of the CMOS focal stack is varied to see its effect on the ranging performance: it can be seen by comparing horizontally the root mean square error (RMSE) in the 2nd, 3rd and 4th columns or in the 5th and 6th columns that higher resolution focal stack gives lower loss. Besides, note that spatially averaged results have lower loss, compared to those without averaging. This is because the noise from interference fringes is suppressed after applying spatial averaging.

	4×4 Graphene	4×4 CMOS	9×9 CMOS	32×32 CMOS	4×4 (Avg. 20) CMOS	9×9 (Avg. 20) CMOS
RMSE x	0.012	0.031	0.020	0.021	0.014	0.009
RMSE y	0.014	0.028	0.017	0.012	0.012	0.010
RMSE z	1.196	1.304	1.192	0.480	0.616	0.458

Supplementary Table 1. Single-point object 3D ranging RMSE (unit: mm) table on testing set. Avg. 20 means spatial averaging with window size 20 is performed on the raw high-resolution focal stack.

	4×4 Graphene	4×4 CMOS	9×9 CMOS	32×32 CMOS	4×4 (Avg. 20) CMOS	9×9 (Avg. 20) CMOS
2p2s	0.017	0.036	0.025	0.013	0.020	0.013
2p3s	0.019	0.033	0.022	0.013	0.019	0.012
3p2s	0.019	0.042	0.027	0.025	0.021	0.016
3p3s	0.021	0.041	0.029	0.028	0.022	0.017

Supplementary Table 2. Multi-point object 3D ranging RMSE (unit: mm) table of x on testing set. Avg. 20 means spatial averaging with window size 20 is performed on the raw high-resolution focal stack. First column encodes different object configurations, e.g., 2p3s means 2-point object with 3 possible shapes.

	4×4 Graphene	4×4 CMOS	9×9 CMOS	32×32 CMOS	4×4 (Avg. 20) CMOS	9×9 (Avg. 20) CMOS
2p2s	0.022	0.045	0.033	0.019	0.026	0.017
2p3s	0.025	0.039	0.028	0.018	0.025	0.015
3p2s	0.010	0.019	0.013	0.016	0.011	0.007
3p3s	0.019	0.035	0.026	0.027	0.021	0.016

Supplementary Table 3. Multi-point object 3D ranging RMSE (unit: mm) table of y on testing set. Avg. 20 means spatial averaging with window size 20 is performed on the raw high-resolution focal stack. First column encodes different object configurations, e.g., 2p3s means 2-point object with 3 possible shapes.

	4×4 Graphene	4×4 CMOS	9×9 CMOS	32×32 CMOS	4×4 (Avg. 20) CMOS	9×9 (Avg. 20) CMOS
2p2s	0.685	1.073	0.759	0.349	0.557	0.371
2p3s	1.164	1.573	1.142	0.788	0.983	0.641
3p2s	0.793	1.328	0.876	0.715	0.750	0.470
3p3s	0.894	1.444	1.004	0.895	0.850	0.594

Supplementary Table 4. Multi-point object 3D ranging RMSE (unit: mm) table of z on a testing set. Avg. 20 means spatial averaging with window size 20 is performed on the raw high-resolution focal stack. First column encodes different object configurations, e.g., 2p3s means 2-point object with 3 possible shapes.

Supplementary Tables 2, 3, 4 summarize the study of the effect of the detector resolution and spatial smoothing on the multi-point object 3D ranging performance. Similar to the single-point object case, more pixels are useful in reducing the ranging error, as can be seen by comparing horizontally the RMSE in 2nd, 3rd and 4th columns or in the 5th and 6th columns. The spatial averaging is again helpful, as in the single object case, in reducing the estimation error.

The numerical results summarized in Supplementary Table 1-4 above are also illustrated graphically in Supplementary Fig. 7-11 below.

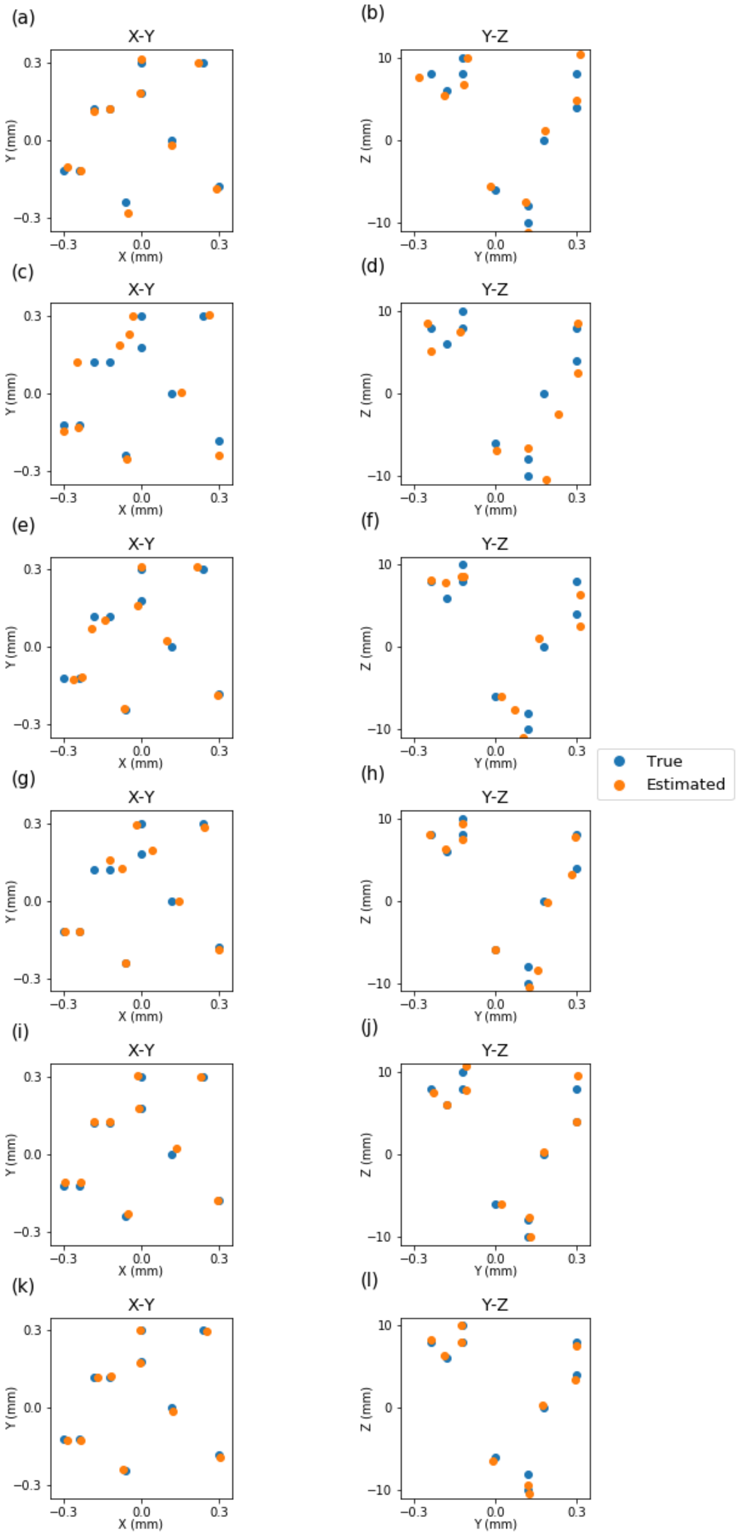

Supplementary Figure 7. Single-point object tracking performance (only 10 test samples are shown). Focal stack data from: (a-b) 4×4 transparent graphene detector. (c-d) 4×4 CMOS sensor. (e-f) 9×9

CMOS sensor. (g-h) 32×32 CMOS sensor. (i-j) 4×4 Avg. 20 CMOS sensor. (k-l) 9×9 Avg. 20 CMOS sensor.

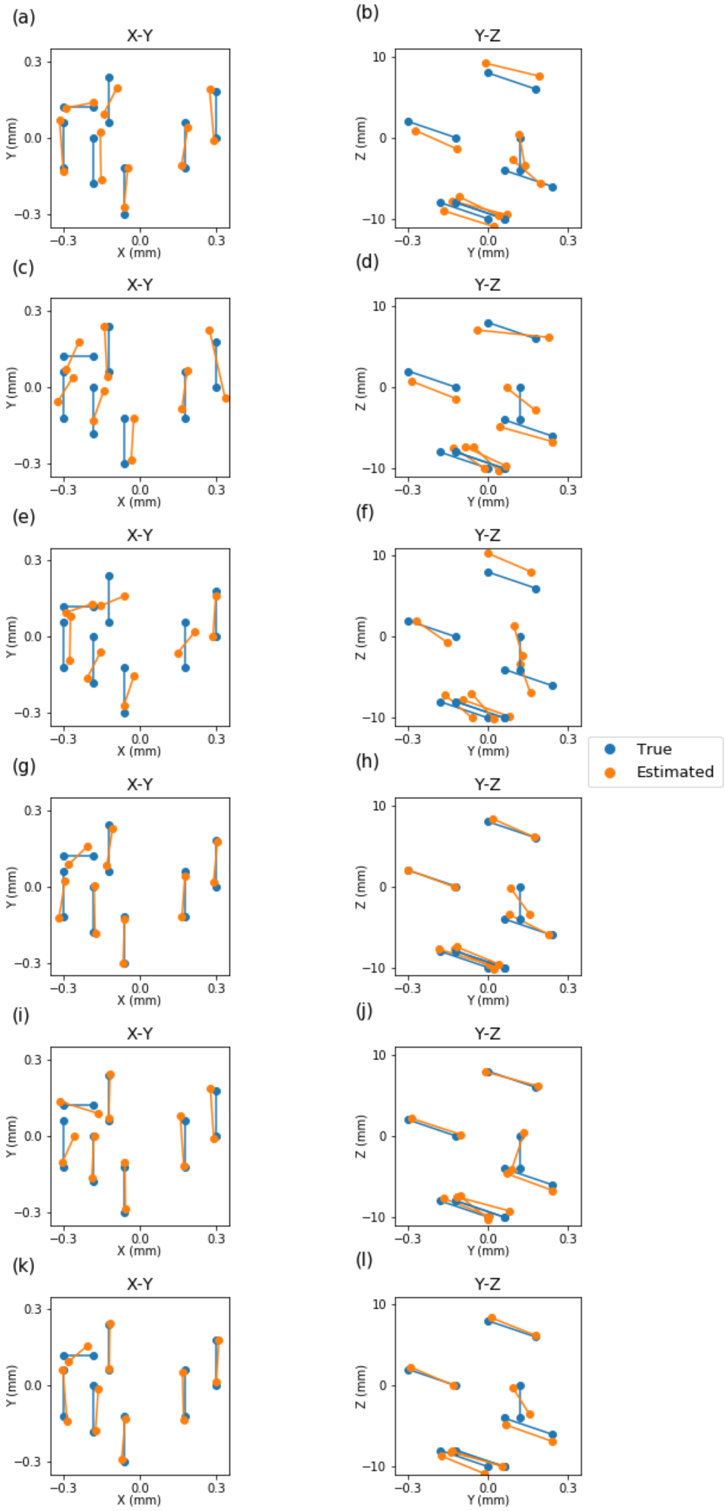

Supplementary Figure 8. 2-point object with 2 possible shapes tracking performance (only 7 test samples are shown). Focal stack data from: (a-b) 4×4 transparent graphene detector. (c-d) 4×4 CMOS sensor. (e-f) 9×9 CMOS sensor. (g-h) 32×32 CMOS sensor. (i-j) 4×4 Avg. 20 CMOS sensor. (k-l) 9×9 Avg. 20 CMOS sensor.

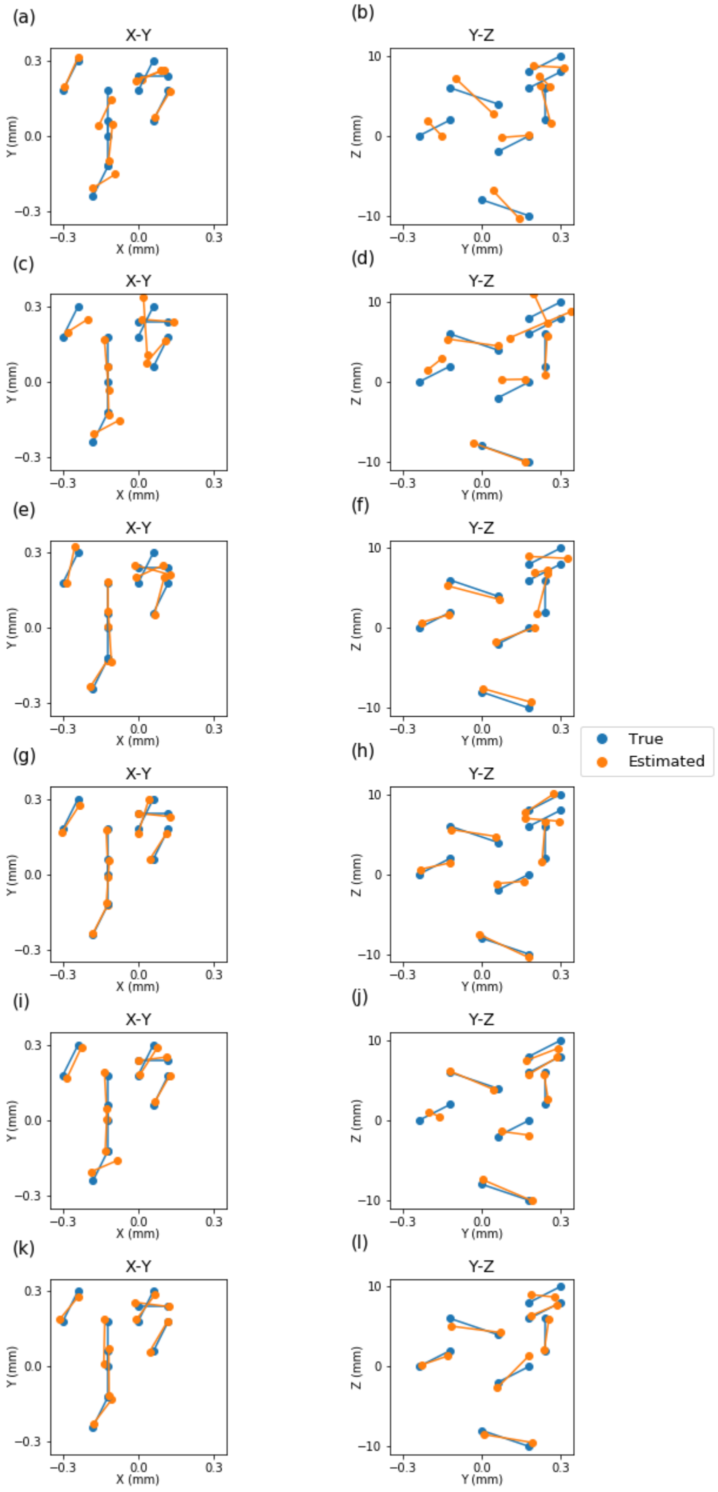

Supplementary Figure 9. 2-point object with 3 possible shapes tracking performance (only 7 test samples are shown). Focal stack data from: (a-b) 4 × 4 transparent graphene detector. (c-d) 4 × 4 CMOS sensor. (e-f) 9 × 9 CMOS sensor. (g-h) 32 × 32 CMOS sensor. (i-j) 4 × 4 Avg. 20 CMOS sensor. (k-l) 9 × 9 Avg. 20 CMOS sensor.

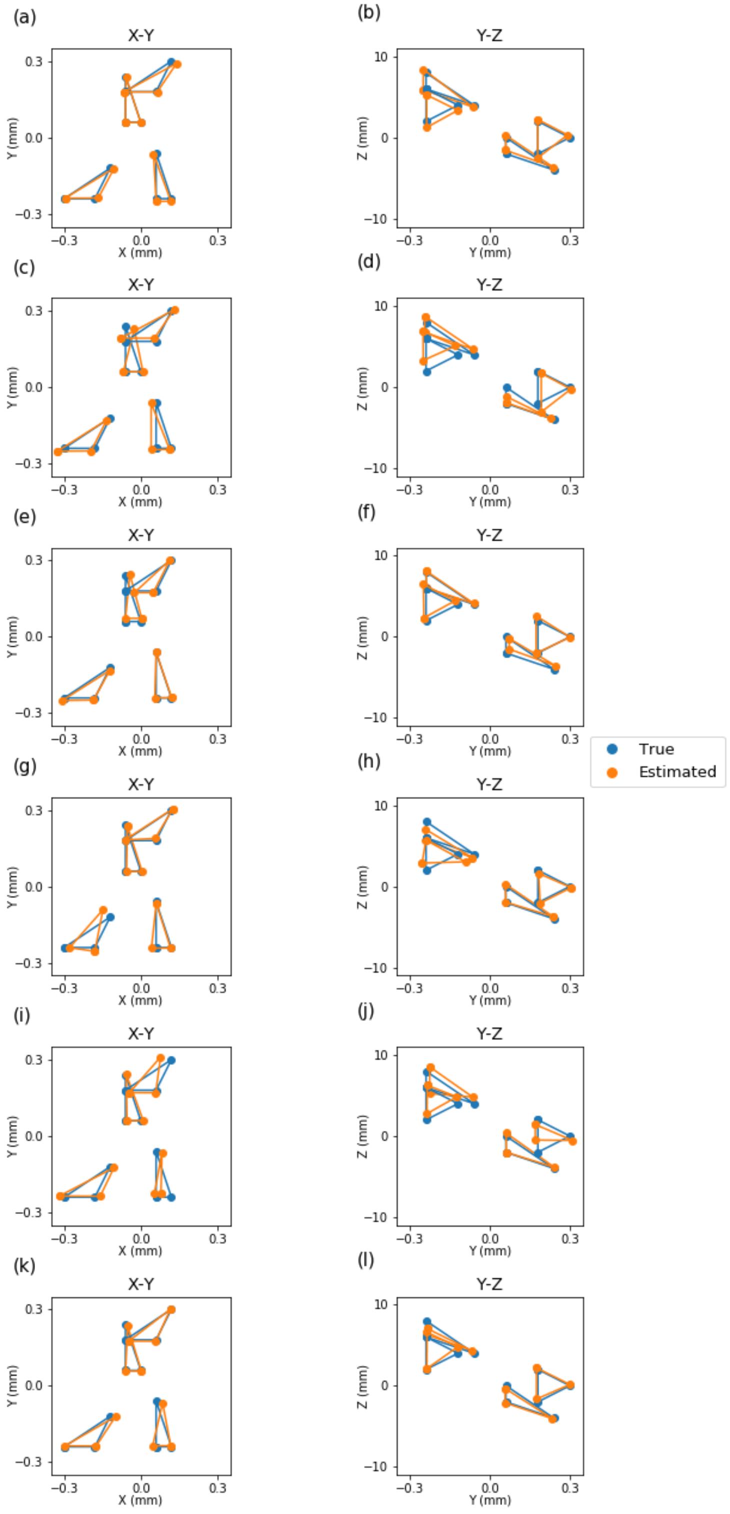

Supplementary Figure 10. 3-point object with 2 possible shapes tracking performance (only 4 test samples are shown). Focal stack data from: (a-b) 4 × 4 transparent graphene detector. (c-d) 4 × 4 CMOS sensor.

(e-f) 9 × 9 CMOS sensor. (g-h) 32 × 32 CMOS sensor. (i-j) 4 × 4 Avg. 20 CMOS sensor. (k-l) 9 × 9 Avg. 20 CMOS sensor.

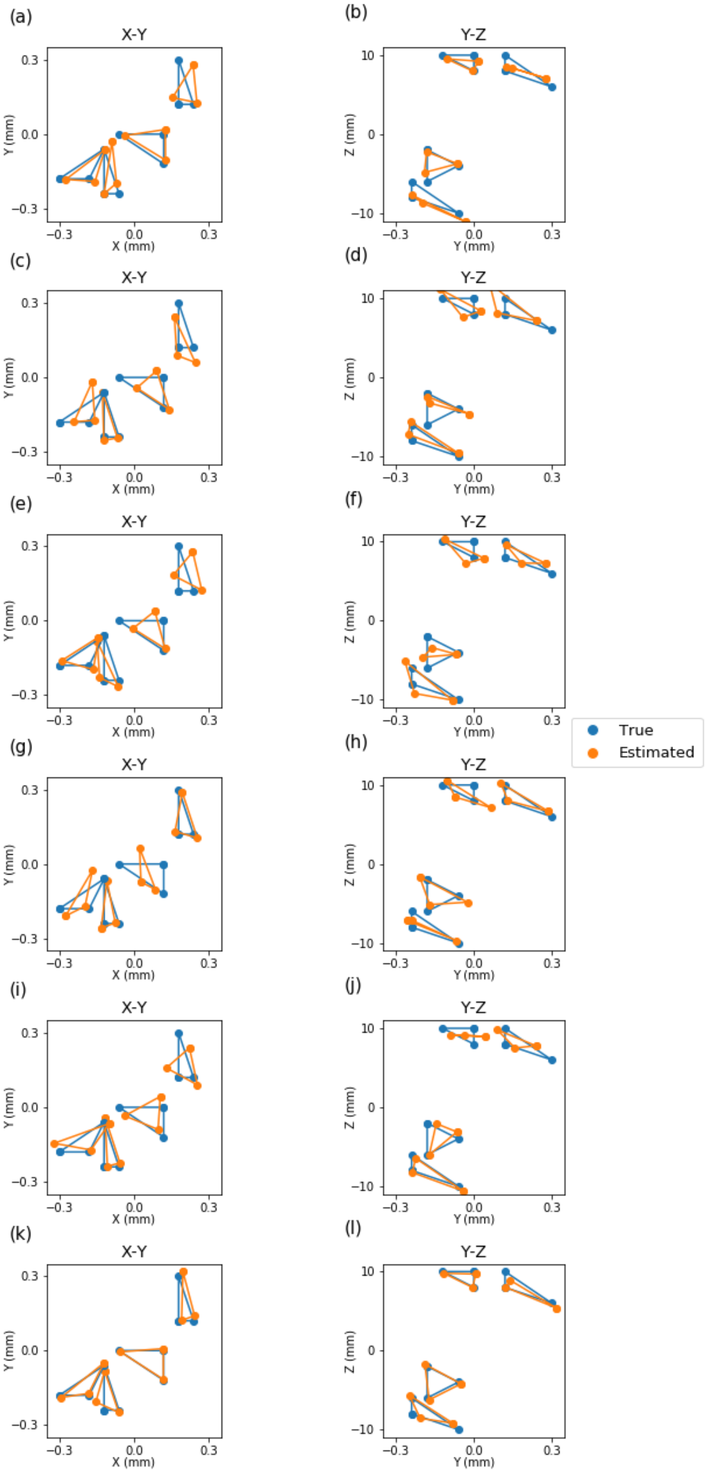

Supplementary Figure 11. 3-point object with 3 possible shapes tracking performance (only 4 test samples are shown). Focal stack data from: (a-b) 4 × 4 transparent graphene detector. (c-d) 4 × 4 CMOS sensor.

(e-f) 9 × 9 CMOS sensor. (g-h) 32 × 32 CMOS sensor. (i-j) 4 × 4 Avg. 20 CMOS sensor. (k-l) 9 × 9 Avg. 20 CMOS sensor.

VI. Inference Time

Supplementary Table 4 shows the network inference time for point object tracking. The inference times for extended object position tracking and orientation estimation are 9.538 ms and 6.001 ms, respectively. We measured the inference time using a 1531 MHz Nvidia GeForce 1080 Ti GPU with 11G RAM.

	4×4	9×9	32×32
Single-point object	0.503 ms	0.512 ms	0.539 ms
2-point object	0.187 ms	0.190 ms	0.192 ms
3-point object	0.190 ms	0.189 ms	0.189 ms

Supplementary Table 4. Point object tracking inference time for different input focal stack resolutions.

Supplementary References:

[1] Lee, Seunghyun, et al. "Homogeneous bilayer graphene film based flexible transparent conductor." Nanoscale 4.2 (2012): 639-644.

[2] Zhang D, Cheng G, Xu Z, et al. Electrically tunable photoresponse in a graphene heterostructure photodetector[C]//2017 Conference on Lasers and Electro-Optics (CLEO). IEEE, 2017: 1-2.

[3] Balandin A A. Low-frequency 1/f noise in graphene devices[J]. Nature nanotechnology, 2013, 8(8): 549-555.

[4] Spietz L, Lehnert K W, Siddiqi I, et al. Primary electronic thermometry using the shot noise of a tunnel junction[J]. Science, 2003, 300(5627): 1929-1932.

[5] F. Rosenblatt, Psychol. Rev. 65, 386 (1958).

[6] Kingma, Diederik P., and Jimmy Ba. "Adam (2014), A method for stochastic optimization." Proceedings of the 3rd International Conference on Learning Representations (ICLR), arXiv preprint arXiv. Vol. 1412.

[7] S. Karen and A. Zisserman, ArXiv:1409.1556v6 (2014).

[8] https://pytorch.org/docs/stable/notes/randomness.html
